# Neuro-Ophthalmic Presentation of Steroid 5a-Reductase Type 3 Congenital Disorder of Glycosylation: A Case of Monozygotic Twins

**DOI:** 10.7759/cureus.102557

**Published:** 2026-01-29

**Authors:** Shikha Swaroop, Sudeshna Dasgupta, Preeti Srivastava, Shikhar Deep Jain, Deb Sanjay Nag, Sanjay K Tanti

**Affiliations:** 1 Pediatrics, Manipal-Tata Medical College, Manipal Academy of Higher Education, Tata Main Hospital, Jamshedpur, IND; 2 Pediatrics, Tata Main Hospital, Noamundi, IND; 3 Anesthesiology, Tata Main Hospital, Jamshedpur, IND

**Keywords:** congenital disorders of glycosylation, hypotonia, nystagmus, optic atrophy, whole exome sequencing

## Abstract

We report a very rare autosomal recessive metabolic disorder in monozygotic twin sisters caused by the steroid 5a-reductase type 3 (SRD5A3) gene defect, a subtype of congenital disorder of glycosylation (CDG). SRD5A3 activity is required for N-glycosylation of proteins. This step is important for the protein to gain its function. The condition is characterized by severe neurodevelopmental delay, cerebellar atrophy or hypoplasia, ocular abnormalities, and ichthyotic skin changes. We describe 20-month-old female monozygotic twins born to non-consanguineous South Asian parents. Notably, both twins exhibited generalized tonic-clonic seizures starting in early infancy - a feature less commonly reported in SRD5A3-CDG. Physical examination of both children showed central hypotonia and bilateral horizontal nystagmus. Fundoscopy of the twins showed optic disc pallor suggestive of optic atrophy. Other features, such as ichthyosis and joint laxity, were absent. Crucially, despite prominent neurological symptoms, brain MRIs at 20 months were entirely normal, showing no evidence of cerebellar hypoplasia or atrophy typically associated with this condition. Whole-exome sequencing identified a homozygous nonsense mutation at exon 1 c.57G>A (p.Trp19Ter), in the SRD5A3 gene, classified as a pathogenic variant as per the American College of Medical Genetics and Genomics (ACMG), helping in establishing the diagnosis. SRD5A3-CDG should be one of the differentials in infants with unexplained seizures, hypotonia, and early ocular signs. This case highlights the phenotypic diversity of SRD5A3-CDG and demonstrates that structural brain anomalies may be absent in the early years of life. It underscores the importance of considering CDG as a differential in infants with unexplained hypotonia and ocular signs, even in the setting of normal neuroimaging.

## Introduction

Congenital disorders of glycosylation (CDGs) are a clinically and genetically diverse group of inherited metabolic disorders characterized by defects in the synthesis or attachment of glycans to proteins and lipids [1]. Steroid 5a-reductase type 3 (SRD5A3) CDG, a rare subtype first identified in 2010, arises from biallelic variants of the SRD5A3 gene [2]. A significant diagnostic challenge lies in the fact that SRD5A3-CDG can present with normal transferrin isoelectric focusing, the standard screening tool for most CDGs, often leading to delayed diagnosis. The SRD5A3 enzyme plays a crucial role in dolichol synthesis, a lipid carrier indispensable for the initial stages of N-linked glycosylation [3]. This essential post-translational modification influences protein folding, stability, and function, thereby affecting several physiological processes [3]. Because the structural integrity of the retina and central nervous system is highly dependent on properly glycosylated proteins, early neuro-ophthalmic signs serve as critical markers for this underlying metabolic defect.

The clinical manifestations of SRD5A3‑CDG are broad and typically encompass severe neurodevelopmental impairment, ocular abnormalities such as optic atrophy and coloboma, ichthyotic skin changes, and cerebellar malformations [1]. We present a case of monozygotic female twins who exhibited global developmental delay, seizures, and bilateral optic atrophy in early infancy. Whole exome sequencing (WES) identified biallelic pathogenic variants in SRD5A3, establishing the diagnosis of SRD5A3‑CDG. This case underscores the significance of early ocular and neurological manifestations in SRD5A3‑CDG and highlights the importance of comprehensive genetic testing, such as WES, in children with nonspecific neurodevelopmental features. This case is unique as it demonstrates a "radiologically silent" presentation of SRD5A3-CDG, where significant neuro-ophthalmic symptoms occurred in the absence of the hallmark cerebellar atrophy or cutaneous changes.

## Case presentation

Patient history

Twenty-month-old monozygotic twins born to non-consanguineous South Asian parents exhibited global developmental delay and a history of seizures with onset during early infancy. The first twin experienced her initial generalized tonic-clonic seizure at six months of age, while the second twin experienced a similar seizure at five months of age. Seizures were generalized tonic-clonic in type, in both. Effective seizure control was achieved with 20 mg/kg/day of sodium valproate. The twins were delivered via caesarean section at 34 weeks of gestation following an uncomplicated pregnancy, with a normal antenatal anomaly scan performed at 20 weeks of gestation. Both twins had no significant family medical history.

Clinical examination

Both twins exhibited global developmental delay. At 20 months, in the motor domain, both children could only sit with support, and in the language domain, only cooing and crying were present. Physical examination of both twins revealed normocephalic children of average build. Anthropometric measurements were appropriate for age. Facial dysmorphism was absent. Neurological evaluation was notable for central hypotonia and persistent bilateral horizontal nystagmus in both twins. Fundoscopic examination in both twins revealed bilateral optic disc pallor, suggestive of optic atrophy. Systemic examination was otherwise unremarkable. On visual assessment, both twins demonstrated poor fixation and a lack of tracking, consistent with the identified optic atrophy. While formal hearing screening was not performed, both infants exhibited age-appropriate behavioral responses to auditory stimuli. A summary of clinical features is presented in Table 1.

**Table 1:** Summary of key clinical features

Clinical features	Twin 1	Twin 2
Head Circumference	Normal	Normal
Dysmorphism	No	No
Seizure Onset	6 Months	5 Months
Seizure Type	Generalized Tonic-Clonic	Generalized Tonic-Clonic
Neurological	Central Hypotonia, Nystagmus	Central Hypotonia, Nystagmus
Ophthalmic Finding	Bilateral Optic Atrophy	Bilateral Optic Atrophy

​​​Investigation

​​​​​​Routine metabolic and endocrine workup (thyroid-stimulating hormone, vitamin D level, kidney function test, and liver function test) was within normal limits. Renal ultrasound (USG KUB) was normal, and 2D echocardiography revealed no cardiac anomalies. Electroencephalography (EEG) and contrast-enhanced magnetic resonance imaging (CEMRI) of brain performed at 20 months were reported as normal for both twins, with no evidence of cerebellar atrophy, hypoplasia, or brainstem abnormalities. Blood tandem mass spectrometry (TMS) and urine gas chromatography-mass spectrometry (GCMS) were normal in both children. A summary of key investigations is presented in Table 2.

**Table 2:** Investigations of both the twins TSH: Thyroid-Stimulating Hormone, SGPT: Serum Glutamic Pyruvate Transaminase, SGOT: Serum Glutamic-Oxaloacetic Transaminase, ALP: Alkaline Phosphatase, KUB: Kidney Ureter Bladder, CEMRI: Contrast-Enhanced Magnetic Resonance Imaging, TMS: Tandem Mass Spectrometry, GCMS: Gas Chromatography-Mass Spectrometry. Reference ranges adapted for age.

Investigation	Twin 1	Twin 2	Units	Reference range
Vitamin D	33.98	30.61	ng/mL	10-50
TSH	4.18	1.73	uIU/mL	0.3-6.0
Urea	31.6	11-36	mg/dL	11-36
Serum Creatinine	0.44	0.2-0.4	mg/dL	0.44
Total Bilirubin	0.26	0.28	mg/dL	0.2-1.2
Direct Bilirubin	0.07	0.06	mg/dL	0.1-0.5
Indirect Bilirubin	0.19	0.22	mg/dL	0.1-0.7
SGPT	14.1	14.4	U/L	0-45
SGOT	36.4	37.6	U/L	0-35
ALP	235.2	232.2	U/L	150-350
Ultrasound KUB	Normal	Normal		
2D Echocardiography	Normal	Normal		
CEMR Brain	Normal	Normal		
Blood TMS	Normal	Normal		
Urine GCMS	Normal	Normal		

Genetic analysis

Given the constellation of neurodevelopmental delay, seizure, and optic atrophy in the absence of significant birth insult or post-natal neurology infection, a genetic etiology was suspected. Whole-exome sequencing (WES) for both twins was done. Analysis identified a homozygous nonsense variant, c.57G>A (p.Trp19Ter), in exon 1 of the SRD5A3 gene in both twins. This variant was classified as pathogenic as per the American College of Medical Genetics and Genomics (ACMG) (Table 3). The p.Trp19Ter mutation is a nonsense variant located early in the first exon, leading to a premature stop codon and a predicted loss of protein function through nonsense-mediated decay. This results in a failure of the SRD5A3 enzyme to catalyze the conversion of polyprenol to dolichol, thereby disrupting the crucial lipid carrier synthesis required for N-linked glycosylation pathways [2].

**Table 3:** Whole exome sequencing report ACMG codes: PVS1 (null variant in a gene where loss of function is a known mechanism), PM2 (absent in population databases), PP5 (variant interpreted as pathogenic by reputable sources).

Gene (Transcript)	Location	Variant	Zygosity	Disease (OMIM)	Inheritance	Classification
SRD5A3 (+) (ENST00000264228.9)	Exon 1	c.57G>A (p.Trp19Ter)	Homozygous	Congenital disorder of glycosylation, type Iq (OMIM#612379)	Autosomal recessive	Pathogenic (PVS1, PM2, PP5)

Genetic counselling was provided to the parents, including a discussion on the recurrence risk and the need for parental evaluation for the carrier state. It was explained to the parents that the parental carrier state risk in future pregnancies could be ascertained.

## Discussion

This case describes the early presentation of SRD5A3-CDG in monozygotic twins, characterized by hypotonia, infantile-onset seizures, optic atrophy, nystagmus, and global developmental delay. The observed phenotype aligns with prior studies that establish hypotonia and ophthalmological features as primary manifestations of SRD5A3-CDG [4-6]. The early onset of nystagmus and optic atrophy in our patients is consistent with the essential role of SRD5A3-mediated glycosylation in the maintenance of retinal proteins [5,6]. The identified homozygous SRD5A3 c.57G>A (p.Trp19Ter) variant is a known recurrent pathogenic mutation in SRD5A3‑CDG, with an allele frequency higher than other variants in South Asians [7,8].

Several differential diagnoses were considered. Mitochondrial encephalopathy was initially suspected because of the combination of seizures and hypotonia, although normal metabolic markers and neuroimaging made this less probable [9]. Leber congenital amaurosis (LCA) was the primary ophthalmological differential diagnosis given the early onset of nystagmus; however, the involvement of multiple systems suggested a glycosylation defect [10]. Finally, the absence of the "molar tooth sign" on MRI ruled out Joubert syndrome [11]. Definitive diagnosis was achieved only through WES, which is increasingly recognized as a first-line tool for complex presentations.

Our patients' phenotypes demonstrated the core neurological and ophthalmological manifestations, particularly early‑onset nystagmus and optic atrophy, linked to defective glycosylation of retinal proteins. However, our case differed from previous reports in that it lacked cutaneous abnormalities (e.g., ichthyosis), joint laxity, and brain abnormalities (e.g., cerebral atrophy and cerebellar hypoplasia) [6]. This suggests broader phenotypic variability, and a normal brain MRI at 20 months indicates that atrophic changes in the brain may not be an early feature. This observation suggests a broader range of phenotypic variability, with a normal brain MRI at 20 months indicating that structural anomalies may not be an early feature. The absence of cerebellar hypoplasia at 20 months in both twins is a significant finding. Although cerebellar atrophy is considered a common feature of SRD5A3-CDG, our case illustrates that functional deficits, such as nystagmus and hypotonia, can manifest prior to the appearance of structural changes on imaging [12]. This "radiologically silent" phase may create a false sense of security and delay diagnosis if clinicians rely solely on classic MRI markers before initiating genetic testing. Onset of seizure at an early age is another distinguishing characteristic in our cases, as seizure has not been commonly described in such disorders [6]. Seizures are a recognized but variably reported feature of SRD5A3-CDG, and their exact prevalence in the literature remains poorly defined [13].

Limitations

The limitations of this case report primarily pertain to the incomplete genetic validation and the challenge of broader applicability. A significant constraint is the incomplete segregation analysis, as the paternal carrier state could not be assessed due to the parents' refusal, thereby hindering full confirmation of the inheritance pattern within this family. Additionally, since the study examines a single pair of monozygotic twins, the findings - particularly the "radiologically silent" brain imaging and infantile uncommon seizures - constitute a unique clinical presentation that may not be readily generalizable to all SRD5A3-CDG patients. The absence of classic indicators, such as ichthyosis and cerebellar atrophy, further underscores the disorder's broad phenotypic variability, suggesting that these results may not serve as universal markers.

## Conclusions

In conclusion, the cases of monozygotic twins with SRD5A3-CDG contribute to the expanding phenotypic spectrum of this rare metabolic disorder. The occurrence of early-onset seizures and the absence of the characteristic cerebellar atrophy at 20 months highlight that the clinical presentation may be "radiologically silent" during early infancy. This report underscores that the p.Trp19Ter nonsense mutation leads to a significant loss of enzyme function, while structural brain changes may not immediately manifest alongside functional neurological and ocular deficits. Clinicians should maintain a high level of suspicion for congenital disorders of glycosylation in patients exhibiting the triad of global developmental delay, hypotonia, and nystagmus, irrespective of whether traditional metabolic screenings or neuroimaging results are abnormal. Ultimately, early neuro-ophthalmic findings may precede structural brain abnormalities and should prompt early genetic evaluation, even when neuroimaging appears unremarkable.
